# Dried blood spot IP-10 as a biomarker of tuberculosis severity and treatment responses: results from a phase IIb trial of adjunctive acetylsalicylic acid and ibuprofen

**DOI:** 10.3389/fimmu.2026.1924239

**Published:** 2026-10-12

**Authors:** Iselin Høgbakk Edlund, Noelia Alonso-Rodriguez, Chiara Sopegno, Judith Farrés, Nino Gogichadze, Lilibeth Arias, Kaori L Fonseca, Kennedy Otwombe, Ziyaad Waja, Tumelo Moloantoa, Natasha Pillay, Thabiso Seiphetlo, Neil Martinson, Cristina Vilaplana, Anne Ma Dyrhol-Riise

**Affiliations:** 1 Department of Infectious Diseases, Oslo University Hospital, Oslo, Norway; 2 Institute of Clinical Medicine, University of Oslo, Oslo, Norway; 3 Experimental TB Unit, Germans Trias i Pujol Research Institute & Hospital (HUGTIP-IGTP), Badalona, Catalonia, Spain; 4 Laboratory of Structural Bioinformatics (GRIB), Department of Medicine and Life Sciences, Universitat Pompeu Fabra, Barcelona, Catalonia, Spain; 5 Universitat Autònoma de Barcelona, Bellaterra, Catalonia, Spain; 6 Centro de Investigación Biomédica en Red de Enfermedades Respiratorias (CIBERES), Madrid, Spain; 7 Perinatal HIV Research Unit (PHRU), University of the Witwatersrand, Johannesburg, South Africa; 8 School of Public Health, Faculty of Health Sciences, University of the Witwatersrand, Johannesburg, South Africa; 9 Centre for TB Research, Johns Hopkins University, Baltimore, MD, United States; 10 Microbiology Department, Northern Metropolitan Clinical Laboratory, Hospital Universitari Germans Trias I Pujol, Badalona, Catalonia, Spain

**Keywords:** aspirin, dried blood spots (DBS), host-directed adjunct therapies, ibuprofen, IP-10 (Interferon-γ-inducible 10 kD protein), point-of-care, tuberculosis

## Abstract

**Background:**

Host-directed therapies may improve tuberculosis (TB) outcomes by modulating immune responses and attenuating hyperinflammation, yet accessible biomarkers remain limited. We hypothesized that interferon-γ–inducible protein 10 (IP-10/CXCL10) measured in dried blood spot (DBS), a low-threshold and field-adapted sampling method, has potential as a biomarker for treatment response in pulmonary TB. Accordingly, we assessed IP-10 levels in patients receiving adjunctive acetylsalicylic acid (ASA) or ibuprofen (IBU) alongside TB standard therapy.

**Methods:**

IP-10 levels were quantified longitudinally in DBS from 120 pulmonary TB patients enrolled in the SMA-TB clinical trial (NCT04575519) at two sites in South Africa. Samples collected at baseline, week 2, week 8, and week 24 were analyzed by the enzyme-linked immunosorbent assay (ELISA). Associations with disease severity and treatment response were assessed, and the predictive value of IP-10 for microbiological, clinical, and radiological outcomes was evaluated.

**Results:**

IP-10 levels correlated with TB disease severity, with higher concentrations observed in patients with elevated clinical and radiological scores and positive sputum acid-fast-bacilli staining. This association was reflected in treatment response, as patients with good clinical outcomes exhibited an earlier decline in IP-10 (week 8, p=0.0005), whereas those with poor outcomes showed a delayed decline (week 24, p<0.0001). Overall, IP-10 levels decreased during treatment irrespective of intervention or HIV status, with no significant differences between treatment arms at week 24. Notably, IP-10 levels at week 2 predicted rapid sputum culture conversion in the IBU arm, as well as in HIV-negative patients in the ASA arm (AUC: ASA 0.79; IBU 0.76).

**Conclusion:**

IP-10 levels measured in DBS are associated with disease severity and treatment response and may serve as a biomarker for therapy monitoring and early prediction of microbiological outcomes in TB, irrespective of adjunctive host-directed therapies. These findings require validation in larger patient cohorts before the method can be implemented in clinical practice.

## 1 Introduction

Tuberculosis (TB) remains a major global health problem with around 10.7 million new cases of active TB and 1.23 million deaths in 2024 (1). Current methods for monitoring treatment responses are associated with several limitations, including long turnaround times for culture results and the limited ability of PCR-based methods to distinguish between viable and non-viable mycobacteria (2–4). Accordingly, there is a substantial need for robust biomarkers and point-of-care (POC) tests that can improve patient monitoring and enable more accurate prediction of treatment outcomes, especially in resource-limited settings with a high burden of TB.

TB treatment includes several antibiotics given over months and is associated with several side effects (5, 6). To shorten therapy, improve outcomes, and prevent TB sequelae, there is growing interest in host-directed therapies (HDTs) adjunctive to standard TB treatment that augment host defence mechanisms or modulate excessive inflammation (7). Different approaches for HDTs targeting distinct mechanisms of the host immune system have been explored (7, 8) and the use of repurposed drugs represents a practical and cost-effective approach. Non-steroidal anti-inflammatory drugs (NSAIDs) such as acetylsalicylic acid (ASA) and ibuprofen (IBU) may minimise tissue damage by inhibiting cyclooxygenase (COX) enzymes and thereby the production of prostaglandins (9). It has been reported both from animal studies and clinical TB trials that NSAIDs are safe and may reduce inflammation, but the need for a personalised treatment strategy has been highlighted (10–13). Still, tailored therapy based on individual patient characteristics are not yet available in routine clinical practice (14, 15).

Interferon-γ (IFN-γ) induces the expression of multiple cytokines and chemokines, including the pro-inflammatory chemokine interferon-γ–inducible protein 10 (IP-10/CXCL10), produced by various immune cell types as part of the inflammatory response (16, 17). IP-10 plays a central role in mediating leukocyte recruitment to sites of infection and is implicated in cell proliferation and angiogenesis, with increased expression observed in a range of viral and bacterial infections (17). IP-10 has been investigated as a biomarker of disease progression and treatment outcomes in infections such as HIV (18) and COVID-19 (19). In TB, IP-10 levels are higher in active disease compared with latent infection and decline with successful treatment (20–25). However, some studies show that IP-10 levels remain higher in people living with HIV (PLWH) compared to TB patients without HIV co-infection (20, 22).

Dried blood spot (DBS), a minimally invasive and field-adapted sampling approach allows easy storage and transport of blood samples on filter paper (26, 27). We have previously demonstrated declining IP-10 levels from DBS during anti-TBs treatment in pulmonary TB cohorts from Norway (20, 25) and in extrapulmonary TB patients from a low-resource setting, including in individuals living with HIV (22). More recently, DBS-based profiling of inflammatory biomarkers in extrapulmonary TB further supports the utility of DBS sampling as a platform for treatment monitoring in resource-constrained settings (28). Despite these encouraging findings, previous DBS-based studies have largely focused on observational cohorts of pulmonary or extrapulmonary TB patients and primarily evaluated biomarker changes during standard anti-TB therapy (22, 25) and not in the context of randomized clinical trials with predefined microbiological, clinical and radiological endpoints. Whether DBS-derived IP-10 can provide information on disease severity, predict predefined treatment outcomes, or retain its utility in the setting of adjunctive HDTs has not previously been investigated.

The SMA-TB clinical trial investigated adjunct NSAIDs treatment in pulmonary TB (29, 30). In this sub-study, we aimed to explore whether IP-10 levels in blood obtained by DBS from patients recruited into the SMA-TB clinical trial could reflect TB disease severity and if DBS-measured IP-10 could be used as a predictive biomarker for outcomes in adjunctive HDT.

## 2 Material and methods

### 2.1 Study participants

The present study includes pulmonary TB patients from the SMA-TB trial (NCT04575519), a randomized, multicentre, phase IIB, placebo-controlled, double-blinded clinical trial evaluating ASA and IBU as adjunctive therapy to standard of care (SoC) TB regimen, conducted from March 2021 to December 2023 (29, 30). A total of 120 South African TB patients; 42, 40 and 38 allocated to the Placebo (PBO), ASA and IBU arms, respectively, were included (**Table 1**). PLWH with CD4 count ≥ 100 cells/mm^3^ and receiving antiretroviral therapy (ART) with viral load ≤ 400 copies/ml at the time of recruitment were included. Routine blood samples were obtained at each visit to analyse haemoglobin (Hb), neutrophil/lymphocyte ratio (N/L), monocyte/lymphocyte ratio (M/L) and erythrocyte sedimentation rate (ESR). For detailed information about inclusion and exclusion criteria, see protocol (29).

**Table 1:** Demographic and clinical characteristics of the study participants at baseline across intervention arms.

Demographics	Placebo	ASA	IBU	P-value
	n = 42 (35.0)	n = 40 (33.3)	n = 38 (31.7)	
Women, n (%)	11 (26.2)	6 (15.0)	13 (34.2)	0.14
Age, years	34 (25-41)	38 (30-45)	37 (33-44)	0.14
Daily smoker, n (%)	16 (38.1)	24 (60.0)	19 (50.0)	0.14
Daily alcohol intake, n (%)	2 (4.8)	4 (10.0)	1 (2.6)	0.39
Clinical characteristics				
PLWH, n (%)	17 (40.5)	16 (40.0)	16 (42.1)	0.98
MDR-TB, n (%)	2 (4.8)	2 (5)	1 (2.6)	>0.99
AFB positive^#^, n (%)	34 (81.0)	35 (87.5)	29 (76.3)	0.46
TB score	4.5 (3.3-6)	5.0 (4-6)	4.0 (2.3-5)	0.16
X-ray AS	10.0 (6-16)	8.0 (6-12)	6.0 (4.3-11.5)	0.07
Cavities, n (%)	34 (81.0)	31 (77.5)	31 (81.6)	0.89
Laboratory parameters				
CD4 count^##^	469 (325-579)	439 (300-692)	329 (176-480)	0.16
Anaemia, n (%) ^###^	27 (64.3)	25 (62.5)	25 (65.8)	0.96
Haemoglobin, g/dL	12.2 (10.9-13.5)	12.8 (11.2-14.0)	12.1 (11.0-13.3)	0.57
Monocyte/Lymphocyte ratio	0.40 (0.32-0.58)	0.42 (0.30-0.64)	0.39 (0.31-0.59)	0.89
Neutrophil/Lymphocyte ratio	2.80 (2.25-4.43)	3.38 (2.50-5.27)	3.19 (1.97-4.72)	0.47
ESR, mm/hr	60 (27-80)	55 (32-76)	51 (24-84)	0.79

Baseline characteristics and demographic data of TB patients. Continuous variables are presented as median with interquartile range. PLWH, People living with HIV; X-ray AS, X-ray Activity Score; AFB, Acid Fast Bacilli; ESR, Erythrocyte Sedimentation rate. #Three patients, one in each intervention arm had unknown AFB at baseline. ##CD4 counts for PLWH only. ###Anemia was defined using cut-off values of 13,5 g/dL for men and 12,0 g/dL for women. TB score was calculated using following parameters: cough, haemoptysis, dyspnoea, chest pain, night sweats, anaemia, pulse > 80 beats/min, positive findings on lung auscultation, temperature > 37 °C, BMI < 18, BMI < 16, MUAC < 220 mm and MUAC < 200 mm (29, 32). Xray AS was calculated using the RUTI-TB Evolution Score (RTBES) (33). The Chi-square test (or Fisher’s exact test for sample sizes) was used to analyse differences across the treatment groups for categorical variables. For continuous variables a Kruskal-Wallis test was used to analyse differences across the treatment groups. Statistical significance was defined as p < 0.05 for all comparisons.

### 2.2 Ethical considerations

The clinical study was approved by the South African Health Products Regulatory Authority (SAHPRA; 20200501), the Human Research Ethics Committee of the University of the Witwatersrand, South Africa (200408B), and the Human Research Ethics Committee of the Hospital Universitari Germans Trias i Pujol, Spain (PI-21-028). Ethical clearance was obtained from the Regional Committee for Medical and Health Research Ethics, Southeast-Norway (REK sør-øst 2022464438). The study was conducted in compliance with the Declaration of Helsinki and in accordance with the International Conference on Harmonisation’s Good Clinical Practices guidelines and is registered in ClinicalTrials.gov (NCT04575519). All participants provided written informed consent. Permission to export DBS samples out of South Africa was approved by the South African Department of Health. Samples were transported from clinical site to biobank at IGTP (Biobank National registry (Collections section) of the ISCIII and the Spanish Ministry of Science and Innovation, C. 0006127) and subsequently stored in the approved biobank at the Department of infectious Diseases, Oslo University Hospital (OUH), Norway (Research Biobank Infectious Disease, REK nr.6.2008.173).

### 2.3 Definitions of clinical severity and outcomes

Severity of TB was defined by microbiological, clinical and radiological parameters as defined by the trial protocol (29, 31). Microbiological response was determined by sustained sputum culture conversion (SCC). Fast responders had SCC ≤ 8 weeks and slow responders SCC > 8 weeks (31). A relapse was defined as a positive culture 6 months after the end of therapy. Patients with positive culture, or only one negative culture by week 24 were defined as non-responders. Clinical severity was determined by the TB score, including symptoms and signs, as previously described (32) (**Table 1**). A good clinical outcome (GC-OUT) was defined as a sustained reduction in TB score ≥ 50% from baseline to week 8 (TBS-50R) and/or ≥ 75% reduction from baseline to week 24 (TBS-75R). Patients who did not experience this reduction in TB score were defined as having a poor clinical outcome (PC-OUT). Radiological severity (chest X-ray Activity Score; X-ray AS) was calculated by the RUTI-TB Evolution Score (RTBES) (33). A good radiological outcome (GR-OUT) was defined as a sustained reduction in the X-ray AS of ≥ 50% from baseline to week 8 (X-ray AS-50R) and/or ≥ 75% from baseline to week 24 (X-ray AS-75R). Patients who did not experience this reduction were defined as having a poor radiological outcome (PR-OUT).

### 2.4 Dried blood spot filter paper sample preparation

Samples from 120 patients, at baseline (BL), week 2 (W2), week 8 (W8) and week 24 (W24) after initiation of therapy were collected at the clinical sites. Blood samples were drawn either directly from a finger pinch to filter paper or into EDTA tubes and pipetted on to Whatman 903 filter paper. Spots were left to dry for 3–4 hours at room temperature (RT) before placed in zip-locked bags with desiccants and stored at −80 °C until shipped by air in RT to OUH, where they were stored at -80 °C until analysis consistent with manufacturers recommendations.

### 2.5 IP-10 analyzed from dried blood spots

IP-10 levels extracted from DBS samples were measured by the enzyme-linked immunosorbent assay (ELISA) using a commercially available ELISA kit for IP-10/CXCL10 (R&D Systems, Cat. no. DIP100), according to the manufacturer’s recommendations. Two 6 mm discs were punched from each patient’s Whatman 903 filter paper, placed horizontally together in a filter microtiter plate, and incubated with 120 µL of Assay Diluent RD1–56 buffer for 1.5 hours on a shaker. The filter microtiter plate was then centrifuged at 700G for 10 minutes. Eluate (40μL) was pipetted to its respective well in the pre-coated ELISA plate hydrated with 40μL of Assay diluent RD1–56 buffer. All samples were processed in duplicates. IP-10 concentrations were calculated from standard curves. Samples falling outside the assay range, as well as those with duplicate measurements showing a coefficient of variation (%CV) greater than 20%, were excluded from statistical analysis. In total, seven samples were excluded, and 450 samples were available for analysis. Between-run assay performance was monitored using an internal control, collected on DBS, included on each plate as duplicates. The between-run coefficient of variation (%CV) was 15% indicating an acceptable inter-assay precision.

### 2.6 Statistical analysis

Statistical analyses were performed in GraphPad Prism software (Graphpad Software, inc., San Diego, CA, USA, version 10.4.1), using non-parametric methods with statistical differences defined as p-values <0.05 (*), <0.01 (**), <0.001 (***) and <0.0001 (****). For descriptive analyses, the median and interquartile range (IQR) were calculated for continuous variable while frequencies were calculated for categorical variables. Differences in categorical variables across intervention arms were assessed using chi-square test, or Fisher’s exact test when sample sizes were small. The Kruskal-Wallis test was conducted to assess differences in continuous variables across intervention arms and in longitudinal IP-10 levels. Dunn’s test was applied as a *post-hoc* test following the Kruskal-Wallis test for longitudinal changes. A two-tailed Mann-Whitney U test was conducted for groupwise comparisons, using Holm-Šídák method to correct for multiple comparisons. Within-subject correlation between IP-10 and clinical and laboratory variable across all time points were assessed using repeated measures correlation (rmcorr) (34), which accounts for the non-independence of repeated observations within individuals. Cross-sectional correlations at individual time points were computed using Spearman’s rank test. P-values were adjusted using Benjamini-Hochberg correction to control the false discovery rate. The correlation coefficient was considered weak if |r| < 0.40, moderate 0.40-0.70 and strong > 0.70.

Prediction analyses were performed using logistic regression classifiers to predict the different outcome groups within a pre-specified stratification by treatment arm and HIV status using IP-10 values. The classifiers were trained using an optimisation method for imbalanced data based on the Matthews Correlation Coefficient (MCC) (35). Multiple classifiers were trained using either the absolute values at a single time point or the relative ratios between baseline and a later time point. To ensure generalizability, stratified k-fold cross-validation was used, with k = 10 where class sizes allowed (giving approximately 90% train and 10% test per fold). Performance metrics were computed by pooling the out-of-fold predictions across folds, rather than averaging per-fold values, which are unstable for small held-out folds. The AUC was obtained from a single ROC curve built on the pooled predicted probabilities, whereas sensitivity and specificity were derived from the pooled class assignments. The decision thresholds were optimised on the training folds using the Matthews correlation coefficient. Markers were pre-specified as acceptable at a pooled cross-validated AUC ≥ 0.70 with sensitivity and specificity above 0.5. Prediction analyses were performed using Python v.3.11.6.

## 3 Results

### 3.1 Characterization of study participants and outcomes

The clinical and demographic characteristics of the TB patients in this sub-cohort, allocated in the Placebo, ASA and IBU arms, are summarized in **Table 1**. Baseline characteristics, including gender, age, HIV status and laboratory parameters as CD4 count, haemoglobin levels, inflammatory cell ratios and ESR were evenly distributed between the intervention arms with no significant differences. At baseline the overall median TB score was 4 and the median X-ray AS was 8. A total of 21 TB patients (Placebo=9, ASA = 9, IBU = 3) had a TB score >4, an X-ray AS >8 and were positive for sputum acid-fast-bacilli (AFB) staining indicating severe disease (**Supplementary Figure S1A**). Most patients had cavitary disease. There were no significant differences in the distribution of these variables across the different intervention arms, although the highest X-ray AS was observed within the Placebo arm (p=0.07). Overall, 68% of patients were fast responders with SCC by W8, two were non-responders and six relapsed (**Table 2**). Good microbiological, clinical and radiological outcomes were comparable between the intervention arms.

**Table 2:** Proportion of patients with good outcomes during treatment across intervention arms.

Microbiological outcomes	Placebo	ASA	IBU	P-value
	n = 42 (35.0)	n = 40 (33.3)	n = 38 (31.7)	
Fast responders, n (%)	27 (64.3)	27 (65)	28 (73.7)	0.66
Slow responders, n (%)	14 (33.3)	12 (30)	9 (23.7)	0.63
Non-responders, n (%)	1 (2.4)	0 (0)	1 (2.6)	
Relapse, n (%)	3 (7.1)	1 (2.5)	2 (5.3)	0.69
Clinical outcomes				
TBS-50R, n (%)	26 (61.9)	26 (65.0)	26 (68.4)	0.83
TBS-75R, n (%)	29 (69.0)	31 (77.5)	24 (63.2)	0.38
Radiological outcomes				
X-ray AS-50R, n (%)	13 (31.0)	12 (30.0)	6 (15.8)	0.23
X-ray AS-75R, n (%)	25 (59.5)	23 (57.5)	18 (47.4)	0.51

Number and percentage of TB patients with good microbiological, clinical and radiological outcomes across Acetylsalicylic-acid (ASA), ibuprofen (IBU) and Placebo (PBO) intervention arms. Good microbiological outcome was defined as fast responders with sustained sputum culture conversion (SCC) ≤ 8 weeks, poor microbiological outcome was defined as slow responders with SCC > 8 weeks. A relapse was defined as a positive culture 6 months after the end of therapy. Patients with positive culture, or only one negative culture by week 24 were defined as non-responders. One patient in ASA arm could not be classified to a microbiological outcome definition as a fast or slow responder. TB score was calculated using the following parameters: cough, haemoptysis, dyspnoea, chest pain, night sweats, anaemia, pulse > 80 beats/min, positive findings on lung auscultation, temperature > 37 °C, BMI < 18, BMI < 16, MUAC < 220 mm and MUAC < 200 mm (29, 32). Good clinical outcome was defined as a sustainable reduction in TB Score ≥ 50% at W8 (TBS-50R), and/or ≥ 75% at W24 (TBS-75R) compared to baseline. X-ray AS was calculated using the RUTI-TB Evolution Score (RTBES) (33). Good radiological outcome was defined as a reduction in the X-ray AS ≥ 50% at W8 (X-ray AS-50R), and/or ≥75% at W24 (X-ray AS-75R) compared to baseline. The Chi-square test (or Fisher’s exact test for sample sizes) was used to analyse differences across the treatment groups for categorical variables. For continuous variables a Kruskal-Wallis test was used to analyse differences across the treatment groups. Statistical significance was defined as p < 0.05 for all comparisons.

### 3.2 Correlations of IP-10 levels with disease severity indicators

IP-10 levels were correlated with haematological variables and clinical and radiological scores reflecting disease severity. A moderate positive within-patient correlation (r ≥ 0.40) was observed for X-ray AS, whereas weak positive correlations (0.20<r < 0.40) were identified for TB score, ESR, N/L ratio, and M/L ratio. Haemoglobin (Hb) demonstrated a weak negative correlation with IP-10 (r = −0.31) (**Figure 1**). Cross-sectional Spearman correlation analyses at individual time points indicated that most correlations were strongest at earlier time points, with correlations generally weakening over time and few remaining statistically significant by week 24 (**Supplementary Figure S2**).

**Figure 1:**
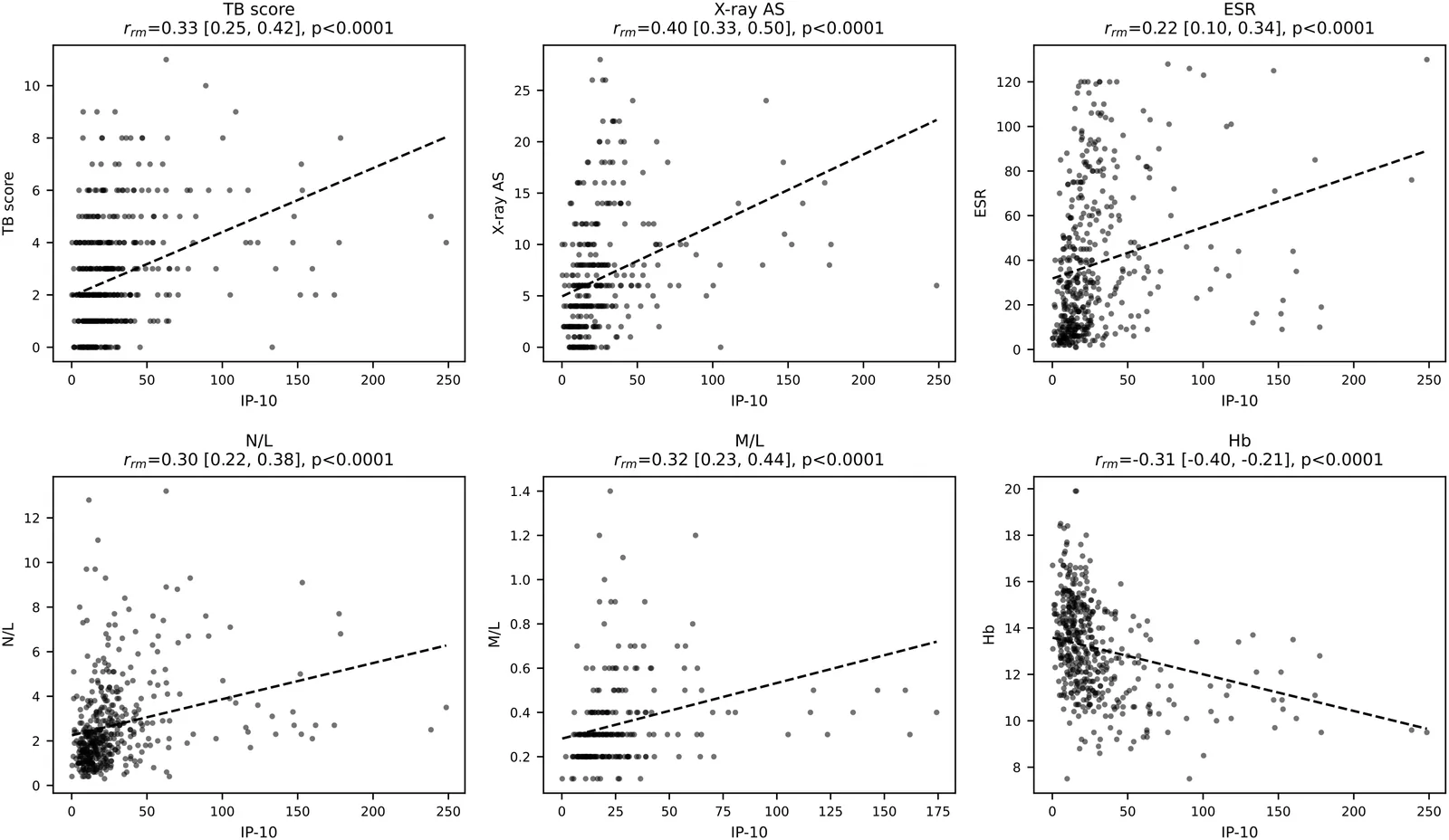
Correlation between IP-10 measured in dried blood spots (DBS) and clinical variables. TB score, X-ray AS, X-ray Activity Score; ESR, erythrocyte sedimentation rate; N/L, neutrophil/lymphocyte ratio; M/L, Monocyte/lymphocyte ratio; Hb, haemoglobin. Correlation analysis included all measured variables at all time points. Repeated measures correlation was used as a statistical method, the rmcorr coefficients (r_rm_) indicate positive correlation and negative correlation, respectively. Correlations coefficient with significant adjusted p-values are presented in figure as scatter plot with each point are individual patient–timepoint observations; the line is the rmcorr common slope, drawn through the overall mean of the points. Panels show r_rm_, 95% CI and p.

### 3.3 Decline in IP-10 during tuberculosis treatment across intervention arms

IP-10 levels were analysed across the intervention arms to evaluate differences due to adjunctive treatment. Patients with TB score and X-ray AS above the cohort’s median value at baseline (TB score >4 and X-ray AS >8), indicating more severe disease, and with positive sputum AFB, had significantly higher IP-10 levels both before and throughout treatment compared to patients with scores below the median value (**Supplementary Figure S1B**).

IP-10 concentration declined over the course of treatment (**Figure 2A**). Although no significant differences were observed between the intervention arms at any time point, significant reductions with respect to baseline were detected in the Placebo (p=0.008) and ASA (p=0.045) arms at W8, whereas in the IBU arm the decline was significant only at W24 (p=0.0001). Of note, longitudinal IP-10 levels showed marked inter-individual variability, with fluctuating trajectories across all intervention arms (**Supplementary Figure S3**).

**Figure 2:**
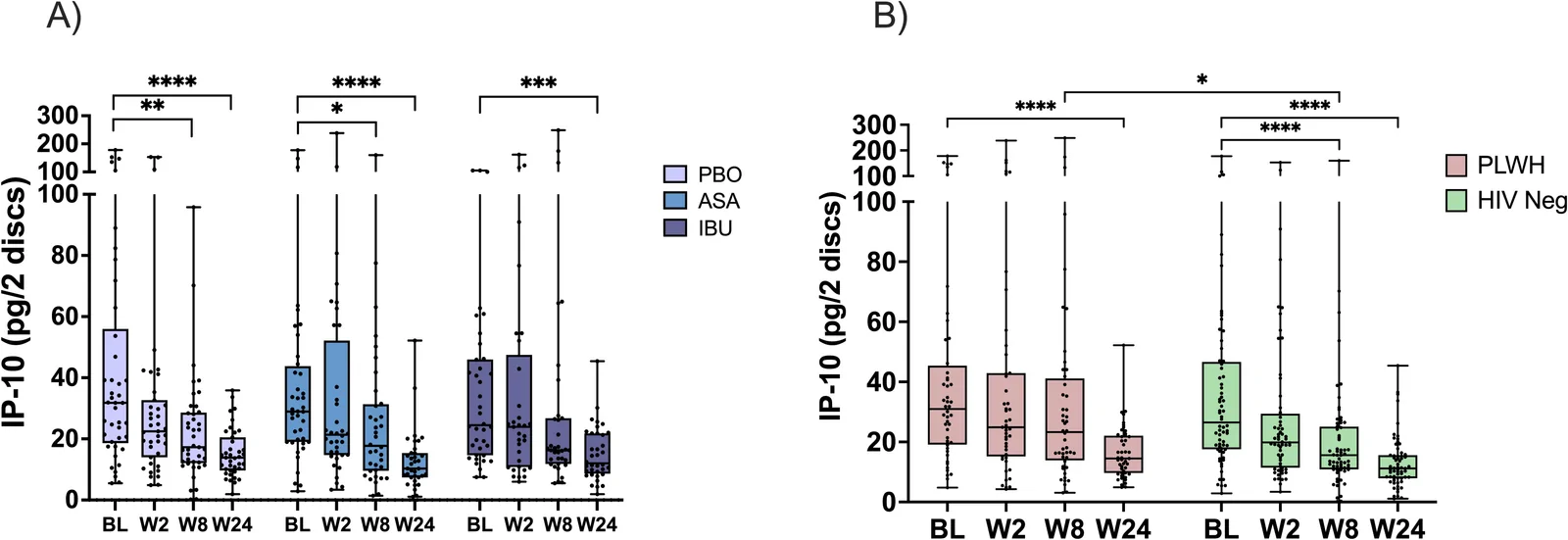
Longitudinal IP-10 levels measured in dried blood spots (DBS) during TB treatment. **(A)** by intervention arms (IBU; Ibuprofen, N = 38, PBO; Placebo, N = 42, ASA; acetylsalicylic-acid, N = 40), and **(B)** by HIV status (PLWH; People living with HIV, N = 49 and HIV negative, N = 70). Box and whisker plots represents median, minimum and maximum values of IP-10 at baseline (BL), week 2 (W2), week 8 (W8), and week 24 (W24). Statistical analysis for longitudinal changes was performed using Kruskal-Wallis, with Dunn’s *post-hoc* analysis. For comparisons according to intervention or HIV status, the Mann-Whitney U-test was performed, using Holm-Šídák method to correct for multiple testing. Significant differences are indicated by asterisks (*p<0.05, **p<0.01, ***p<0.001, ****p<0.0001).

Since IP-10 levels could be influenced by HIV infection, we further examined the longitudinal changes of IP-10 levels in TB patients stratified by HIV status, irrespective of treatment arm (**Figure 2B**). IP-10 levels declined significantly in both PLWH (p<0.0001 at W24) and in HIV negative TB patients (p<0.0001 at W8 and W24). However, the decline occurred earlier in HIV-negative patients, who also exhibited significantly lower IP-10 levels at W8 compared with PLWH (p = 0.040). Still, by the end of therapy at week 24, IP-10 levels were comparable with no significant difference between the two groups.

### 3.4 Early IP-10 decline as a marker of favourable clinical outcome

We explored the longitudinal IP-10 levels across the microbiological (**Figure 3A**), clinical (**Figures 3B, C**) and radiological (**Figures 3D, E**) outcome groups. A significant decline from baseline to W8 was observed in both fast and slow responders, with no differences between the response groups (**Figure 3A**). IP-10 levels were also comparable between the groups at all time points with respect to clinical and radiological outcomes. Still, a significant decline in IP-10 was evident as early as W8 in the GC-OUT group (TBS-50R, p=0.0005), whereas a more gradual decline was observed in the PC-OUT group (week 24, p<0.0001) (**Figures 3B, C**). In contrast, the dynamics of IP-10 were comparable between the GR-OUT and PR-OUT groups, as both demonstrated a significant decline in IP-10 levels by W8 (X-ray AS-50R, p=0.040, X-ray AS-75R, p=0.004) (**Figures 3D, E**).

**Figure 3:**
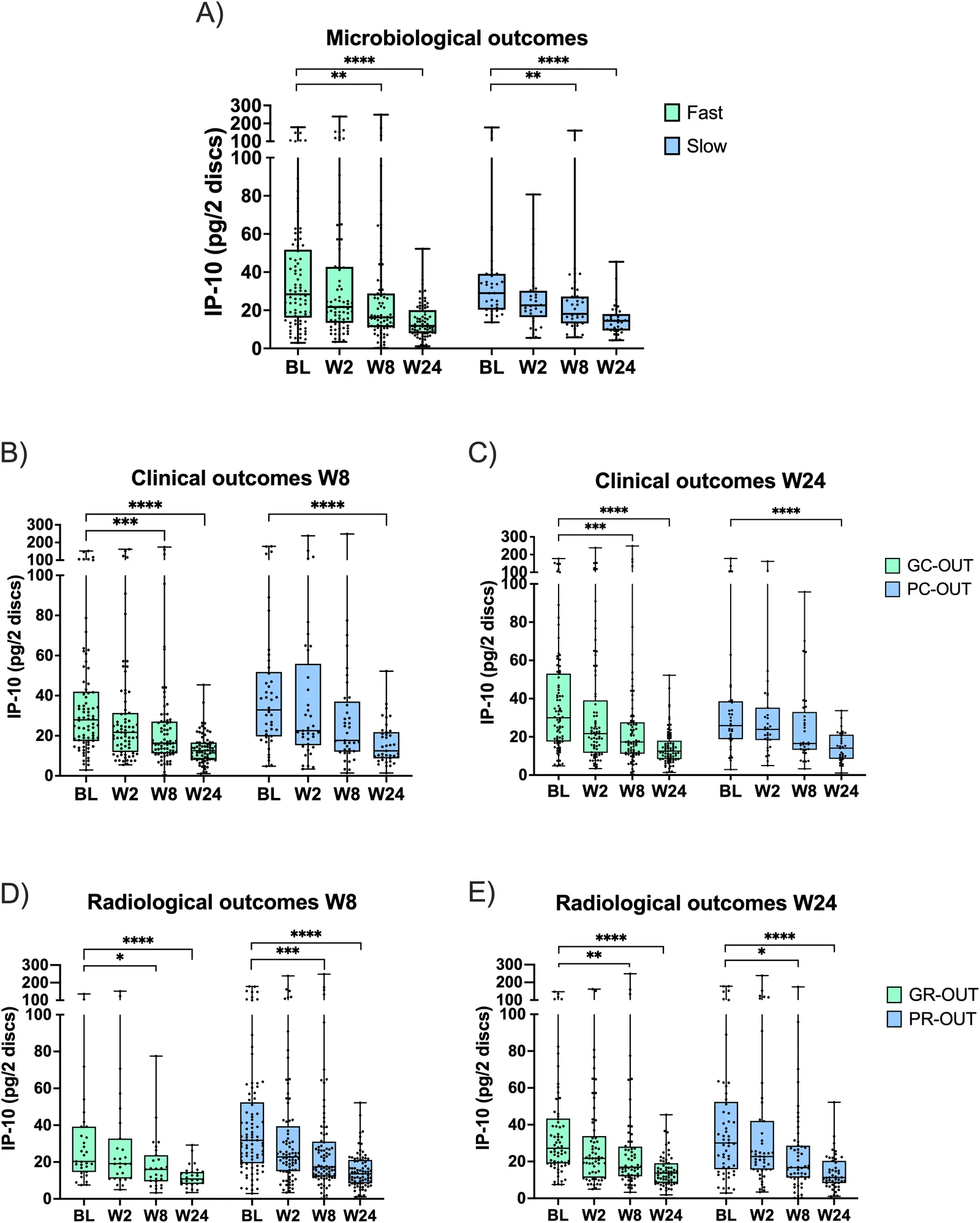
Longitudinal changes of IP-10 across microbiological **(A)**, clinical **(B, C)** and radiological **(D, E)** outcome groups. BL; Baseline, W2; Week 2, W8; Week 8, W24; Week 24. Fast responders were defined as SCC (Sputum Culture Conversion) ≤ 8 weeks and slow responders if SCC > 8 weeks. Good clinical outcomes (GC-OUT) were defined as a ≥ 50% TB Score reduction at W8 (TBS-50R, N = 78) **(B)** and ≥ 75% TBS score reduction at W24 (TBS-75R, N = 84) **(C)**. Good radiological outcomes (GR-OUT) defined as a ≥ 50% X-ray activity score (X-ray AS) reduction at W8 (N = 31) **(D)** and ≥ 75% X-ray AS reduction at W24 (N = 66) **(E)**. Poor clinical outcomes (PC-OUT) and Poor radiological outcomes (PR-OUT) did not reach these criteria. IP-10 levels are represented in box and whisker plots. Statistical analysis for longitudinal changes was performed using Kruskal-Wallis, with Dunn’s *post-hoc* analysis, and for comparisons according to good and poor outcome, the Mann-Whitney U-test was performed, using Holm-Šídák method for correction for multiple testing. Significant differences are indicated by asterisks (*p<0.05, **p<0.01, ***p<0.001, ****p<0.0001).

When comparing outcome groups within each intervention arm, no differences in IP-10 levels were observed at baseline or W2 for Placebo and ASA (**Supplementary Figure S4**). However, within the IBU arm fast responders had higher levels of IP-10 at W2 than slow responders (p=0.04) (**Supplementary Figure S4B**). In addition, patients with poor radiological outcomes (PR-OUT) at W8 exhibited higher baseline IP-10 levels at baseline compared to those with good radiological outcomes (GR-OUT) (**Supplementary Figure S4G**).

### 3.5 Dried blood spots IP-10 as predictive marker for microbiological, clinical and radiological outcomes

As significant differences in IP-10 levels were observed at early time points between good and poor responders with respect to microbiological and radiological outcomes, we next evaluated the predictive potential of IP-10 at baseline, W2, and W8 across intervention arms, with additional stratification by HIV status (**Supplementary Table**). Predictors threshold for acceptable performance was set to equal or higher of 0.70 cross-validated AUC (**Table 3**). Corresponding ROC-curves are presented in **Supplementary Figure S5**.

**Table 3:** Predictive potential of IP-10 for different outcomes across intervention arms.

Outcome	Intervention	Time point	Patient group	AUC (95% CI)	Sens. (95% CI)	Spec. (95%CI)	Threshold (pg/2disks)
Fast responder	Placebo	W8	HIV negative	0.71 (0.48-091)	0.57 (0.30-0.83)	0.8 (0.50-1.00)	<12.57
	ASA	W2	HIV negative	0.79 (0.53-0.99)	0.73 (0.50-0.93)	0.8 (0.33-1.00)	<19.88
	IBU	W2	All	0.76 (0.52-094)	0.75 (0.57-0.91)	0.88 (0.57-1.00)	>19.59
TBS-75R	IBU	W8	PLWH	0.71 (0.40-0.95)	0.75 (0.40-1.00)	0.57 (0.20-1.00)	>16.25
X-ray AS-50R	ASA	W2	HIV negative	0.79 (0.56-0.97)	1 (1.00-1.00)	0.6 (0.35-0.85)	<19.58
X-ray AS-75R	IBU	W2	HIV negative	0.74 (0.46-0.96)	0.75 (0.40-1.00)	0.6 (0.27-0.90)	<12.50

Fast responders (Sputum Culture Conversion < 8 weeks), 50% TB Score reduction at W8 (TBS-50R), 75% TB Score reduction at W24 (TBS-75R), 50% RTBES Activity score reduction at W8 (Xray AS-50R), 75% RTBES Activity Score reduction at W24 (Xray AS-75R), stratified by intervention arms (ASA: acetylsalicylic-acid, IBU: ibuprofen) and HIV status. The table displays those groups for which IP-10 levels showed cross-validated area under the curve (AUC) values ≥ 0.70 and cross-validated sensitivity (Sens.) and specificity (Spec.) > 50%.

IP-10 levels at W2 predicted a fast microbiological response in the IBU arm and similarly in the ASA arm among HIV-negative patients. In contrast, in the Placebo arm, IP-10 levels at W8 predicted a fast response, but only in HIV-negative patients. IP-10 levels at W8 predicted good clinical outcome at the end of therapy (week 24) exclusively in PLWH receiving IBU, with no predictive value observed in the Placebo or ASA arms. For radiological outcomes, IP-10 levels at W2 predicted good response at W8 in the ASA arm, whereas prediction of end-of-therapy radiological outcome was observed in the IBU arm, both restricted to HIV-negative patients.

## 4 Discussion

We present data on blood IP-10 extracted from DBS obtained from pulmonary TB patients recruited into the clinical phase IIB SMA-TB trial on the adjunctive HDTs IBU and ASA compared with standard TB treatment alone (29, 30). This is, to our knowledge, the first study to evaluate DBS-measured IP-10 as a biomarker of disease severity and treatment response within such a trial. We show that DBS IP-10 concentration correlated with a range of clinical variables and was predictive of selected microbiological, clinical, and radiological outcomes in specific patient subgroups.

IP-10 levels declined consistently during efficient TB treatment regardless of interventions with adjunctive HDT or HIV co-infection supporting its robustness as a universal biomarker of treatment response across diverse patient populations. This is consistent with previous studies of pulmonary TB patients receiving standard anti-TB treatment and in patients with extrapulmonary TB, including PLWH (20, 22, 25). Furthermore, DBS-based monitoring of inflammatory host biomarkers has recently been shown to be feasible in extrapulmonary TB, supporting the broader utility of DBS sampling for treatment monitoring (28). In a study of extrapulmonary TB patients in Zanzibar, Hoel et al. showed higher levels of IP-10 in PLWH compared with HIV negative patients (22), whereas in our cohort the IP-10 levels were comparable between the groups. It is important to note that our TB cohort had a low clinical TB score at trial entry that could explain the moderate reduction in IP-10 levels during the first two weeks in contrast to what we have previously reported (25). Still, in this cohort, the decline in IP-10 levels occurred earlier in patients with favourable clinical outcomes with a more rapid resolution of disease burden than in those with poorer outcomes, further supporting IP-10 as a dynamic marker of treatment response. No significant differences in IP-10 levels were observed between treatment arms. However, the timing of the within-group decline differed slightly, with statistically significant reductions detected at week 8 in the Placebo and ASA arms and at week 24 in the IBU arm.

To date, few clinical trials have investigated the effects of NSAIDs during TB treatment. Mortensen et al. demonstrated in murine models that COX inhibition is associated with reduced IFN-γ production by CD4+ T cells (36). Given that both ASA and IBU act as COX inhibitors with anti-inflammatory properties, a concomitant reduction in pro-inflammatory mediators such as IP-10 might be anticipated. Thus, the predictive value of early IP-10 levels for rapid microbiological clearance, improvement in clinical symptoms and reduction in radiological severity was assessed separately within each treatment arm. The most consistent and clinically relevant predictions, characterized by broader applicability and earlier time points, were observed in the IBU and ASA arms were IP-10 levels measured after two weeks of treatment predicted fast sputum conversion. Additionally, early IP-10 levels were predictive of favorable radiological outcomes after 8 weeks in the ASA arm and at the end of therapy in the IBU arm. Although, blood IP-10 levels were modestly correlated with markers of disease severity, the positive correlation with the radiological severity score suggests that higher IP-10 levels reflect greater pulmonary involvement. IP-10 is closely linked to inflammatory activity at sites of infection through IFN-γ-driven immune activation and recruitment of inflammatory cells and may therefore act as a surrogate marker of pulmonary disease activity (16, 17). In contrast, the weaker correlations with TB score, ESR, haemoglobin, and leukocyte ratios imply that IP-10 may better capture local inflammatory activity than conventional clinical scores or blood-based markers. The finding that both high and low IP-10 levels after two weeks of treatment predicted favorable outcomes in different treatment arms underscores the biological heterogeneity of treatment responses. High IP-10 blood levels may reflect the initial host inflammatory response to treatment and thereby capture differences in disease resolution before these become detectable by conventional clinical or microbiological measures. Still, this interpretation remains hypothetical and should be interpreted with caution.

Our findings should be interpreted in the context of the overall SMA-TB trial results (30). Although adjunctive NSAID treatment was not associated with improved microbiological outcomes in the main cohort, radiological analyses suggested earlier cavity resolution in participants receiving ASA or IBU. Thus, the largely comparable IP-10 trajectories we observe across treatment arms are consistent with the absence of major differences in microbiological outcomes, while the associations observed between IP-10 and radiological outcomes warrant further investigation into whether IP-10 may reflect inflammatory processes linked to structural lung recovery.

We and others have previously reported on the potential of using IP-10 measured in a range of biological specimens such as urine (37), stimulated blood (38, 39) and from DBS (22, 25, 40) in both adults and children (41, 42) as a biomarker for TB diagnosis and treatment monitoring. The potential of assessing IP-10 levels in different biological samples and methods facilitates the development of non-sputum, non-invasive diagnostic tools (43), which could contribute to fulfil the World Health Organization’s (WHO) end TB strategy (44). Currently, IP-10-based lateral flow assays developed by independent research groups and commercial manufacturers are undergoing early evaluation, supporting the feasibility of IP-10 as a future POC biomarker (45, 46).

Still, IP-10 as a proinflammatory chemokine is not a specific marker for TB and has also been evaluated as a biomarker for other infections such as Covid-19 (19), HIV (18, 47), hepatitis B (48, 49) and hepatitis C (50). Several patients in our study, irrespective of HIV status, exhibited fluctuating IP-10 levels during therapy consistent with previous reports linking such variability to intercurrent inflammatory conditions (22, 25). Although the TB patients included were considered otherwise healthy, unrecognized intercurrent infections may have influenced IP-10 levels. This represents an important limitation for future clinical implementation, as fluctuations in IP-10 may reflect concurrent infections or other inflammatory conditions rather than TB activity alone. Consequently, IP-10 is unlikely to be sufficiently specific as a stand-alone diagnostic or monitoring tool and should be interpreted together with clinical, microbiological and radiological findings. However, the high sensitivity of IP-10 to inflammatory activity may also be advantageous for treatment monitoring, including in immunocompromised individuals such as PLWH, in whom conventional immune-based biomarkers may perform less well. Thus, future translation may benefit from combining IP-10 with other host biomarkers to improve disease specificity and robustness and further large-scale validation and policy evaluation are still required before routine implementation (2, 51, 52).

We acknowledge that the limited statistical power to identify differences between subgroups is an important limitation in our study. Despite analysing a substantial number of 120 patients from the SMA-TB clinical trial, stratifying into HIV status, treatment arms and outcomes subgroups resulted in smaller sample sizes and the study was not sufficiently powered to assess possible sex-specific differences in immune responses that could potentially influence IP-10 dynamics. Although extensive efforts were made to minimize overfitting through cross-validation of all analyses, this provides only an internal estimate of model performance and cannot exclude overfitting in small subgroups. Accordingly, these findings should be considered hypothesis-generating and require validation in an independent external cohort. Such validation would apply each classifier unchanged to an independent cohort with the same outcome and stratification definitions and would be considered supportive only where a marker reproduced the pre-specified performance criteria. In addition, the cohort size and limited number of outcome events precluded robust development and evaluation using completely independent training and test datasets. Furthermore, the study was not designed to formally compare the predictive performance of IP-10 with conventional inflammatory biomarkers. Such comparative analysis should be addressed in future studies to better define its added clinical value. Also, with few non-responding or relapsing patients, we could not evaluate DBS-measured IP-10 as a predictor for treatment failures as previously reported from IP-10 in serum (52). Finally, while we demonstrate that IP-10 can be reliably detected in DBS filter paper and considered stable under relevant transport conditions supporting its applicability as a robust and field-adapted sampling method in resource-limited settings, the potential pre-analytical effects of unmonitored temperature fluctuations cannot be excluded. Also, the ELISA-based assay used in this proof-of-concept study was not evaluated for cost-effectiveness.

In conclusion, our findings demonstrate a progressive decline in DBS-measured IP-10 levels throughout both standard TB therapy and adjunctive HDT, with an earlier and more pronounced reduction observed in patients with favourable clinical outcomes. Furthermore, elevated IP-10 levels were associated with more severe radiological findings, suggesting a higher and sustained inflammatory burden in these patients compared to those with less extensive lung pathology. Finally, our results suggest that DBS-measured IP-10 may have potential as a predictive biomarker for selected microbiological, clinical, and radiological outcomes in specific patient groups, but further validation is needed before the method can be implemented in clinical practice.

## Data Availability

The datasets presented in this study can be found in online repositories. The names of the repository/repositories and accession number(s) can be found below: https://doi.org/10.5281/zenodo.20914230, 10.5281/zenodo.20914230.
