# Supplementary material for: Dried blood spot IP-10 as a biomarker of tuberculosis severity and treatment responses: results from a phase IIb trial of adjunctive acetylsalicylic acid and ibuprofen
Source: Front Immunol. 2026 Oct 12;17:1924239. doi: 10.3389/fimmu.2026.1924239 (PMC13696322; doi:10.3389/fimmu.2026.1924239)
Supplement: Supplementary file 1 [file SupplementaryFile1.docx]

Supplementary Material

IP-10 Dried Blood Spots as Biomarker of Tuberculosis Severity and Treatment Response: Insights from a Phase IIB Trial of Adjunctive Acetylsalicylic Acid and Ibuprofen

# 1 Supplementary Figures and Tables

## 1.1 Supplementary Figures

**Supplementary Figure S1: Overlap of severity score classification and association to IP-10 levels. A)** Venn diagram illustrating the overlap of patients with higher TB severity at baseline, defined as positive sputum smear microscopy for acid-fast bacilli (AFB), TB score over the median value at BL (TB score > 4), and/or X-ray activity score over the median value at BL (X-ray AS > 8). **B)** Longitudinal changes of IP-10 across low and high TB severity. High TB severity patients (N=21) defined with high TB score, positive AFB and high X-ray AS at baseline, compared with AFB positive TB patients (N=25) with low TB score and low X-ray AS at baseline. Significant differences are indicated by asterisks (*p<0.05, **p<0.01, ***p<0.001, ****p<0.0001).

**Supplementary Figure S2: Correlation between IP-10 measured in DBS and clinical variables during treatment.** TB score, X-ray AS; X-ray Activity Score, ESR; erythrocyte sedimentation rate, N/L; neutrophil/lymphocyte ratio, M/L; Monocyte/lymphocyte ratio, Hb; haemoglobin. Cross-sectional correlations at individual time points were computed using Spearman’s rank test visualized in scatter plot with 95% Confidence interval shown as gray. P-values were adjusted using Benjamini-Hochberg correction to control the false discovery rate.

**Supplementary Figure S3: Longitudinal IP-10 levels with individual trajectories during treatment across intervention arms**. Spaghetti plots illustrating individual patient’s trajectories for IP-10 measured in DBS during treatment, stratified by intervention arms (PBO; Placebo, IBU; Ibuprofen, ASA; acetylsalicylic acid). Each line corresponds to one patient.

**Supplementary Figure S4: IP-10 levels at baseline and W2 across microbiological, clinical and radiological outcome groups, compared within each intervention arm**. BL; Baseline, W2; Week 2, PBO; Placebo, IBU; Ibuprofen, ASA; acetylsalicylic acid. Fast responders defined as Sputum Culture Conversion (SCC) ≤ 8 weeks and slow responders if SCC was > 8 weeks (**A-B**). Good clinical outcomes (GC-OUT) defined as a ≥ 50% TB Score reduction at W8 (TBS-50R) (**C-D**) and ≥ 75% TBS score reduction at W24 (TBS-75R, N=84) (**E-F**). Good radiological outcomes (GR-OUT) defined as a ≥ 50% X-ray AS reduction at W8(**G-H**) and ≥ 75% X-ray AS reduction at W24 (**I-J**). Poor clinical outcomes (PC-OUT) and Poor radiological outcomes (PR-OUT) did not reach these criteria. IP-10 levels are represented in box and whisker plots. Statistical analysis comparisons according to good and poor outcome Mann-Whitney U-test was performed, using Holm-Šídák method for correction for multiple testing. Significant differences are indicated by asterisks (*p<0.05).

**Supplementary Figure S5: Pooled out-of-fold ROC curve for each reported classifier reported in Table 3. (A-C)** ROC curves for predicting fast responders, defined as Sputum Culture Conversion (SCC) ≤ 8 weeks and slow responders if SCC was > 8 weeks, shown separately for the Placebo (PBO) arm (**A**), acetylsalicylic acid (ASA) arm (**B**) and ibuprofen (IBU) arm (**C**). ROC curve for predicting good clinical outcome (GC-OUT) defined as a ≥ 75% TBS score reduction at W24 (TBS-75R W24) in IBU arm (**D**). Good radiological outcomes (GR-OUT) defined as a ≥ 50% X-ray AS reduction at W8 shown in ASA arm (**E**) and ≥ 75% X-ray AS reduction at W24 shown in IBU arm (**F**).
